# Culture time of vitrified/warmed zygotes before microinjection affects the production efficiency of CRISPR-Cas9-mediated knock-in mice

**DOI:** 10.1242/bio.025122

**Published:** 2017-04-10

**Authors:** Yoshiko Nakagawa, Tetsushi Sakuma, Norihisa Nishimichi, Yasuyuki Yokosaki, Toru Takeo, Naomi Nakagata, Takashi Yamamoto

**Affiliations:** 1 Center for Animal Resources and Development, Kumamoto University, 2-2-1 Honjo, Chuo-ku, Kumamoto 860-0811, Japan; 2 Department of Mathematical and Life Sciences, Graduate School of Science, Hiroshima University, 1-3-1 Kagamiyama, Higashi-Hiroshima, Hiroshima 739-8526, Japan; 3 Cell-Matrix Frontier Laboratory, Health Administration Center, Hiroshima University, 1-2-3 Kasumi, Minamiku, Hiroshima 734-8551, Japan; 4 Clinical Genetics, Hiroshima University Hospital, 1-2-3 Kasumi, Minamiku, Hiroshima 734-8551, Japan

**Keywords:** Knock-in, Microinjection, CRISPR-Cas9, Zygote, Culture time, Single-stranded oligodeoxynucleotide (ssODN)

## Abstract

Robust reproductive engineering techniques are required for the efficient and rapid production of genetically modified mice. We have reported the efficient production of genome-edited mice using reproductive engineering techniques, such as ultra-superovulation, *in vitro* fertilization (IVF) and vitrification/warming of zygotes. We usually use vitrified/warmed fertilized oocytes created by IVF for microinjection because of work efficiency and flexible scheduling. Here, we investigated whether the culture time of zygotes before microinjection influences the efficiency of producing knock-in mice. Knock-in mice were generated using clustered regularly interspaced short palindromic repeats (CRISPR)-CRISPR-associated protein 9 (Cas9) system and single-stranded oligodeoxynucleotide (ssODN) or PITCh (Precise Integration into Target Chromosome) system, a method of integrating a donor vector assisted by microhomology-mediated end-joining. The cryopreserved fertilized oocytes were warmed, cultured for several hours and microinjected at different timings. Microinjection was performed with Cas9 protein, guide RNA(s), and an ssODN or PITCh donor plasmid for the ssODN knock-in and the PITCh knock-in, respectively. Different production efficiencies of knock-in mice were observed by changing the timing of microinjection. Our study provides useful information for the CRISPR-Cas9-based generation of knock-in mice.

## Abstract (precis)

**Summary:** We report variable production efficiencies of CRISPR-Cas9-mediated knock-in mice depending on a series of microinjection timings using vitrified, warmed, and cultured zygotes created via ultra-superovulation and *in vitro* fertilization.

## INTRODUCTION

The technique of microinjection into zygotes is essential for the production of genetically modified (GM) mice. During the last five decades, the methods and techniques of pronuclear microinjection have been developed and improved mainly for the production of transgenic mice, and in this time many strains have been generated (Lin, 1966; Jaenisch, 1976, 1988; Gordon et al., 1980; Palmiter et al., 1982; Hanahan, 1989). Transgenes microinjected into zygotes are usually integrated into the genome randomly; therefore, the gene targeting approach based on spontaneous homologous recombination (HR) using embryonic stem (ES) cells was applied for the generation of targeted GM mice (Capecchi, 2005). However, programmable nuclease-mediated genome editing technology enables the direct production of GM mice without using ES cells (Aida et al., 2014). Thus, the microinjection technique has become essential for the production of not only transgenic mice but also genome-edited mice. In recent years, many GM mice have been generated by microinjection of the clustered regularly interspaced short palindromic repeats (CRISPR)-CRISPR-associated protein 9 (Cas9) system (Singh et al., 2015; Kato and Takada, 2017).

CRISPR-Cas9 comprises a complex of Cas9 and a single guide RNA (gRNA), which induces DNA double-strand breaks (DSBs) at the desired genomic locus. After the introduction of a DSB in the target site, it is mainly repaired by non-homologous end-joining (NHEJ). When the repair template [such as a single-stranded oligodeoxynucleotide (ssODN) containing heterologous sequence flanked by homology arms] is co-injected, the heterologous sequence is incorporated via homology-directed repair (HDR). Furthermore, CRISPR-Cas9 can facilitate HR-mediated gene knock-in when a donor plasmid harboring long homology arms (>1 kb) is used. Recently, an alternative gene knock-in strategy, the PITCh (Precise Integration into Target Chromosome) system, was developed (Nakade et al., 2014; Sakuma et al., 2016). In this system, DSBs are repaired by microhomology-mediated end-joining (MMEJ) utilizing very short microhomologies (≤40 bp) instead of long homology arms. The PITCh system has been used for efficient gene knock-in in various cells and organisms (Nakade et al., 2014; Sakuma et al., 2015; Hisano et al., 2015; Aida et al., 2016). Especially, highly practical gene cassette knock-in and floxed mouse generation were reported using a cloning-free CRISPR/Cas system (Aida et al., 2015), a combination of recombinant Cas9 protein and chemically-synthesized dual RNA, and an improved CRISPR-Cas9-mediated PITCh [CRIS-PITCh (v2)] system coupled with exonuclease 1 (*Exo1*) overexpression (Aida et al., 2016). Thus, genome editing technology can introduce precisely defined modifications into a targeted locus, assisted by various DSB repair machineries.

We and other investigators have confirmed that fertilized oocytes created by *in vitro* fertilization (IVF) are applicable for microinjection to create genome-edited mice (Nakagawa et al., 2014, 2015, 2016; Li et al., 2014; Miura et al., 2015; Tsuchiya et al., 2015; Nakao et al., 2016; Sakurai et al., 2016). Recently, we have developed an ultra-superovulation method to collect more oocytes from female mice compared with conventional superovulation methods (Takeo and Nakagata, 2015), and we demonstrated that zygotes created via ultra-superovulation combined with IVF are also applicable for the production of various GM mice by direct microinjection of genome editing reagents (Nakagawa et al., 2016).

**Fig. 1.**
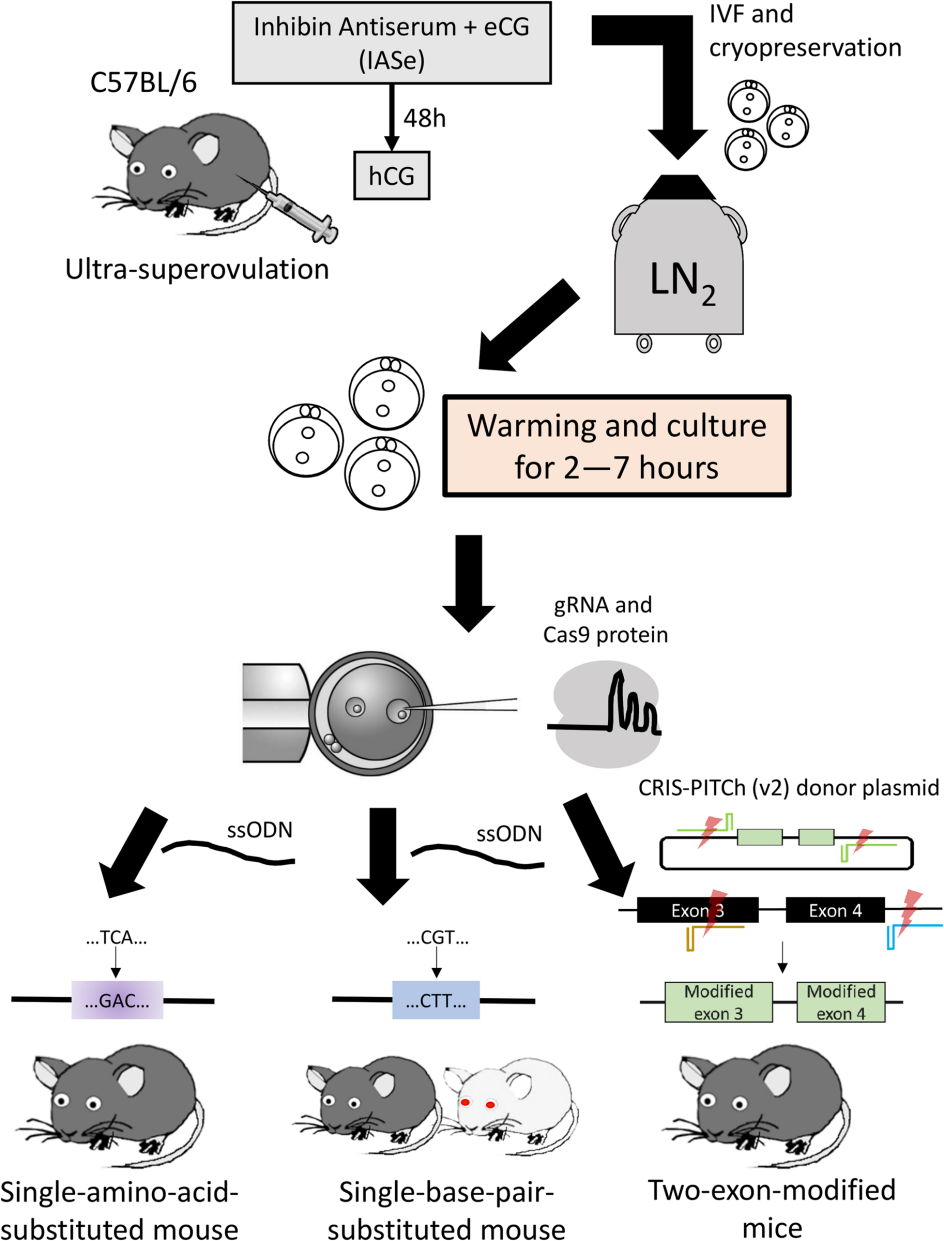
**Schematic overview of the study.** Fertilized oocytes were produced by IVF via ultra-superovulation of female mice and then cryopreserved. After warming, oocytes were cultured for 2–7 h. Subsequently, microinjection was performed using Cas9 RNPs with ssODNs or PITCh donor plasmid. Three kinds of genetically modified mice were generated.

Based on these various technological improvements, a protocol for the CRISPR-Cas9-mediated generation of GM mice is well established. However, there remains room for improvement in terms of the production efficiency of GM mice, especially for knock-in mice. Here, we report CRISPR-Cas9-mediated generation of knock-in mice with various microinjection timings. Using *in vitro* transcribed gRNA(s) and Cas9 protein with either ssODN or CRIS-PITCh (v2) donor plasmid, we generate three kinds of knock-in mice by microinjection into zygotes that were created by IVF, vitrified, then warmed and cultured for 2–7 h (Fig. 1). This study reports an optimized method for creating knock-in mice using vitrified/warmed and cultured zygotes.

## RESULTS

### Generation of mice with a single amino acid substitution at the *Spp1* locus using an ssODN

To investigate whether different culture times of fertilized oocytes lead to different efficiencies of generating knock-in mice, we performed microinjection of Cas9 ribonucleoprotein (RNP) and ssODN carrying the intended substitutions after vitrification/warming and culture of zygotes.

**Fig. 2.**
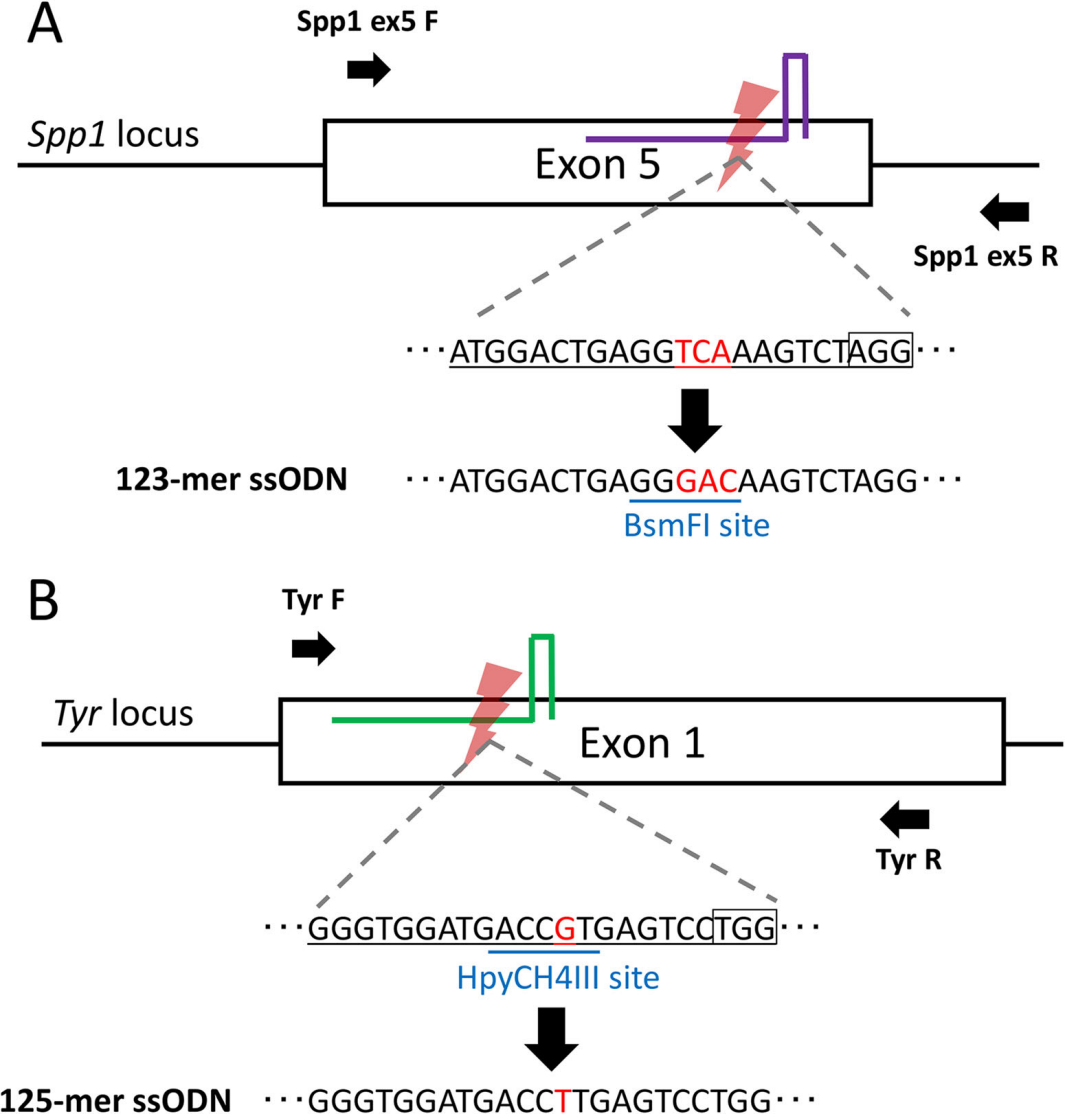
**Generation of base-substituted mice at the *Spp1* and *Tyr* loci.** (A) Schematic illustration to generate a three-base-substituted allele at the *Spp1* locus. A serine residue in exon 5 was replaced with an aspartic acid residue (TCA to GAC; red letters). A gRNA was designed to cut in close vicinity of the serine residue (underlined in black and red). An ssODN was designed to carry the three-base substitution. (B) Schematic illustration to generate a one-base-substituted allele at the *Tyr* locus. A guanine in exon 1 was changed to thymine (red letter). An ssODN was designed to carry the one-base substitution. Black boxes indicate the protospacer adjacent motif (*PAM*) sequences. Arrows indicate the primer sets for PCR. Blue underlined sequences indicate the recognition sites of restriction enzymes for the RFLP analyses.

First, we generated founder mice harboring a three-base substitution that generates a *Bsm*FI recognition site in the secreted phosphoprotein 1 (*Spp1*) gene as we previously reported (Fig. 2A) (Nakagawa et al., 2016). The three-base substitution in exon 5 of *Spp1* was designed to encode a mutant thrombin cleavage-incompetent osteopontin protein (Nishimichi et al., 2011). We warmed cryopreserved fertilized oocytes and then cultured for 2–7 h before microinjection. Partial results of ultra-superovulation, IVF, vitrification/warming, and microinjection were shown in Table S1. We injected Cas9 RNP and ssODN and then transferred the surviving zygotes to pseudopregnant female mice. Overall, restriction fragment length polymorphism (RFLP) analysis using *Bsm*FI and direct sequencing analysis revealed that 17 pups had knock-in alleles (Table 1; Fig. S1). Interestingly, based on the culture time, relatively short (2 h) or long (∼7 h) culture times resulted in high percentages of RFLP-positive pups, whereas intermediate culture times resulted in low percentages, although a low birth rate was observed in the 2 h-cultured zygotes (Table 1).

**Table 1.**
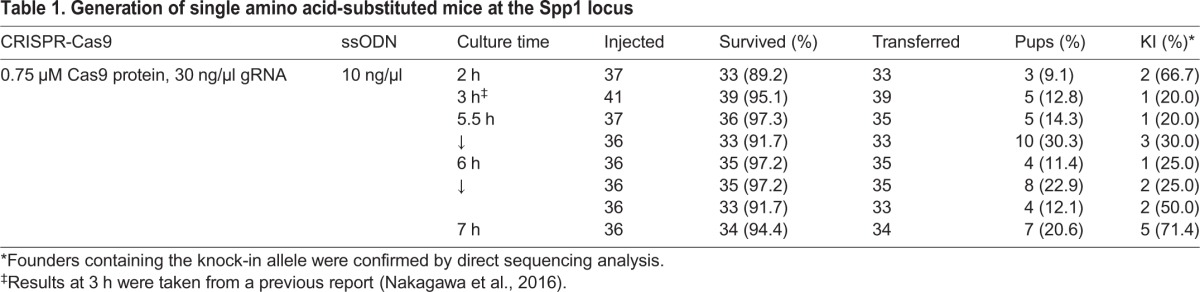
**Generation of single amino acid-substituted mice at the Spp1 locus**

### Generation of mice with a single base-pair substitution at the *Tyr* locus using an ssODN

Next, single-base-pair substitution at another gene locus, tyrosinase (*Tyr*), was examined to investigate the reproducibility of the results observed at the *Spp1* locus (Fig. 2B). We designed a gRNA and ssODN harboring a point mutation (G→T), similar to those in the report of Mizuno and colleagues (2014). This mutation leads to albinism of C57BL/6 mice because the nonfunctional tyrosinase protein causes lack of melanin synthesis. Vitrified/warmed zygotes were cultured for 2–7 h and used for microinjection using Cas9 RNP and ssODN carrying the intended mutation, resulting in an *Hpy*CH4III-resistant allele. Subsequently, the surviving zygotes were transferred and pups were obtained. By using RFLP and direct sequence analyses, the number of precise knock-in pups was determined as 11 (Table 2; Fig. S2). In this case, the RFLP analysis cannot confirm the existence of the intended substituted allele, because simple indel mutations independent of the ssODN donor can also result in the *Hpy*CH4III-resistant allele. Consistent with the results at the *Spp1* locus, fewer pups were born from 2 and 2.5 h cultured zygotes compared to the other culture times, whereas higher rates of correctly targeted pups were observed (Table 2). After culture for 4.5–6 h and 7 h, the frequency of knock-in was low and intermediate, respectively.

**Table 2.**
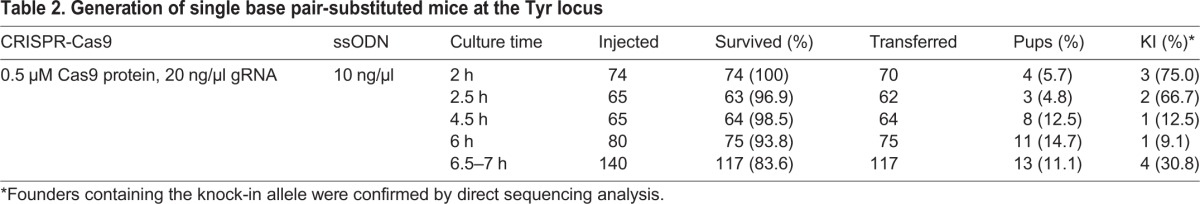
**Generation of single base pair-substituted mice at the Tyr locus**

Overall, base substitutions mediated by ssODNs were favorably introduced in zygotes cultured for 2 or 7 h before microinjection.

### Simultaneous introduction of multiple base pair substitutions using the CRIS-PITCh (v2) system

**Fig. 3.**
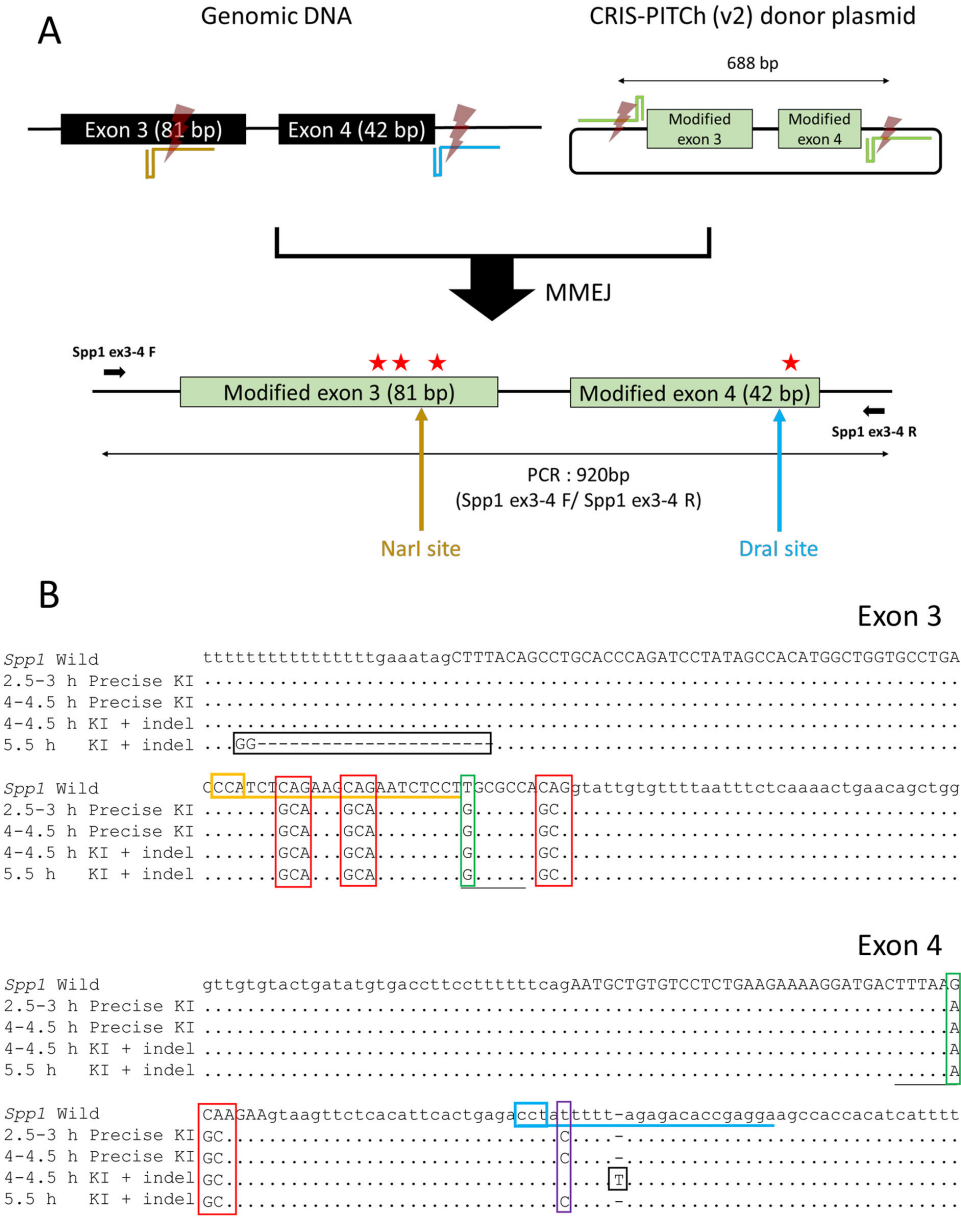
**Generation of knock-in mice at the *Spp1* locus.** (A) Schematic illustration to generate a PITChed allele at the *Spp1* locus, mediated by the CRIS-PITCh (v2) system. Four glutamine residues in exons 3 and 4 (three in exon 3 and one in exon 4) were replaced with alanine residues (red stars). Two gene-specific gRNAs were designed within exon 3 and downstream of exon 4. A PITCh donor plasmid was designed to carry the substituted sequences encoding four alanine residues, silent mutations for RFLP analysis (from T to G in exon 3 and from G to A in exon 4) and a point mutation (from T to C) in intron region. Bold black arrows indicate primers for PCR. Yellow and blue arrows indicate the recognition sites of restriction enzymes for the RFLP analyses. (B) Sequence analysis of subcloned PCR products from pups harboring the knock-in allele. The sequences around exon 3 and exon 4 are displayed in the upper and lower panels, respectively. The modified codons encoding four alanines are enclosed in red boxes. The silent mutations for RFLP analyses are enclosed in green boxes. The gRNA-blocking mutation is enclosed in a purple box. The wild-type allele is shown at the top (*Spp1* Wild) with the gRNA target sequences (underlined in yellow and blue). The PAM sequences are enclosed in yellow and blue boxes. Uppercase letters indicate exon sequences. Dots indicate the same bases as the wild-type sequence. Dashes indicate deletions. Unintended mutations are enclosed in black boxes. Black underlined sequences indicate *Nar*I and *Dra*I sites in exons 3 and exon 4, respectively.

Based on the results of ssODN-mediated knock-in at the *Spp1* and *Tyr* loci, we further investigated whether the culture time of zygotes could affect knock-in efficiency in PITCh knock-in, which uses MMEJ-directed plasmid as a donor. Before microinjection, zygotes were cultured for several hours similar to the ssODN knock-in experiments. Subsequently, we performed microinjection of CRIS-PITCh (v2) donor vector and Cas9 RNP containing two gene-specific gRNAs and one generic gRNA targeting the donor vector for simultaneous modifications of exons 3 and 4 of *Spp1* (Fig. 3A). Four amino acid substitutions spanning the two exons were designed to encode a polymerization-incompetent osteopontin mutant protein (Nishimichi et al., 2011). In addition, we added silent mutations resulting in *Nar*I and *Dra*I sites in exons 3 and exon 4, respectively, for the RFLP genotyping, and a gRNA-blocking mutation in the intronic region, which is a single nucleotide polymorphism naturally found in BALB/c mice, suggesting a functionally inert polymorphism (Fig. 3B). After microinjection, surviving zygotes were transferred to pseudopregnant female mice. Tail lysates of all pups were initially screened using PCR and two RFLP analyses using *Nar*I and *Dra*I. Pups identified as positive for either *Nar*I- or *Dra*I-RFLP or both RFLPs were further analyzed by direct sequencing of the PCR amplicons. Subcloned sequencing or genotyping of F1 pups was subsequently performed to confirm the sequence of knock-in alleles. As summarized in Table 3, three pups and one pup were obtained for 2.5–3 h and 4–4.5 h culture conditions, respectively, that contained all the substituted sequences without any mutations to the knock-in allele. In addition, one pup was obtained for each of the 4–4.5 h and 5.5 h culture conditions that contained the substituted sequences with unintended mutations in the same allele. Collectively, shorter culture times resulted in high knock-in efficiency with the PITCh strategy, which is consistent with the ssODN knock-in results, whereas longer culture times did not result in successful knock-in, unlike the ssODN knock-in.

**Table 3.**
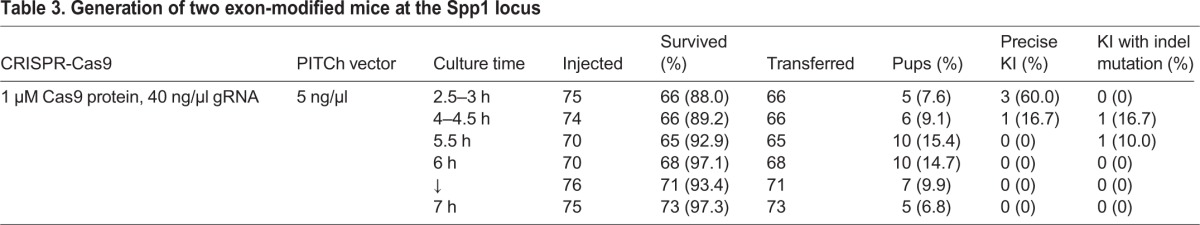
**Generation of two exon-modified mice at the Spp1 locus**

### Transgene analysis and off-target analysis

Because we used plasmid donor DNA for microinjection in the CRIS-PITCh (v2) system, we investigated whether the vector backbone was accidentally integrated into the genome. In six knock-in founders, described above, PCR amplification of the backbone sequence was performed to detect genomic integrants. In two founders, the vector fragment was amplified (Fig. S3); thus, these two founders carried the transgene. However, this might not be a problem, because the transgene can be removed by mating in the next generation.

Finally, we checked for potential off-target sites by direct sequencing in the six PITCh knock-in founder mice. The top five candidate sites were selected using the COSMID web tool (Cradick et al., 2014). PCR amplification around the five target sites in the six founders was performed and the PCR products were sequenced, but we detected no off-target mutations (Table S2).

## DISCUSSION

We have previously described various methods for producing GM mice utilizing reproductive engineering techniques to provide practically useful and efficient protocols for GM mouse generation (Nakagawa et al., 2014, 2015, 2016). Reproductive engineering techniques, such as IVF and vitrification/warming of zygotes allow flexible scheduling of microinjection. In this study, we showed that culture time of zygotes affected the production efficiency of knock-in mice. We generated single-amino-acid-substituted, single-base-pair-substituted, and two-exon-modified mice by microinjecting CRISPR-Cas9 RNPs with ssODN or CRIS-PITCh (v2) donor plasmid into fertilized oocytes cultured for 2–7 h after warming. For base substitutions using an ssODN, we could generate knock-in mice from zygotes cultured for any length of time (between 2 and 7 h) but with high efficiency for short and long culture times and low efficiency for intermediate culture times. In contrast, we succeeded in the production of knock-in mice using only zygotes cultured for a short time, when applying the CRIS-PITCh (v2) system.

Recently, high-efficiency generation of gene knockout and knock-in mice was reported, in which electroporation of CRISPR-Cas9 RNPs with or without an ssODN was conducted (Hashimoto et al., 2016). Although a detailed examination of electroporation timings was not performed by Hashimoto et al., electroporation in zygotes with earlier timing resulted in low mosaicism when introducing NHEJ-mediated mutations. Based on their findings and our investigation, introduction of genome editing reagents in a short period of time after fertilization may contribute to earlier DSB formation, followed by corresponding DSB repairs, resulting in higher gene knockout or knock-in efficiency. On the other hand, our observation of highly efficient ssODN knock-in but unsuccessful PITCh knock-in with 7 h culture conditions might depend on the cell cycle of the injected zygotes. Previous reports suggest that zygotes are in G1 to S phases during our microinjection timings (2–7 h) (Howlett, 1986; Moore et al., 1996). Therefore, zygotes after culture for 7 h can be hypothesized to be around late S phase, where HDR is active and MMEJ is inactive (Taleei and Nikjoo, 2013), thus resulting in successful ssODN knock-in and unsuccessful PITCh knock-in. Further studies are needed to confirm this hypothesis. In addition, the rate of mosaicism is also practically important to establish the knock-in strains. We have not yet analyzed the difference of mosaicism depending on various culture times; thus, it should be clarified in the future study.

Microinjection into zygotes has been carried out in many facilities using the CRISPR-Cas9 system and ssODNs to arrange the target genome for the addition of small modifications, including point mutations, several base-pair substitutions, and insertion of tag sequences; however, the knock-in efficiency was variable (Wang et al., 2013; Wang et al., 2015; Yang et al., 2013; Ran et al., 2013; Fujii et al., 2014; Li et al., 2014; Inui et al., 2014; Han et al., 2015; Mianné et al., 2016; Nakagawa et al., 2016). Although knock-in efficiency can depend on various factors including the target locus, DSB-inducing activity of the gRNA, microinjection conditions, and genetic background of mice used for microinjection, we speculate that microinjection timing varies in each facility. In particular, the developmental stage of zygotes created by mating varies because of differences in the timing of mating and fertilization in each individual, which makes it difficult to control the microinjection timing. In contrast, creating fertilized oocytes using the IVF method enables microinjection timing to be precisely controllable, because many zygotes can be prepared at the same developmental stage. The timing of fertilization is almost equal to that of insemination, and zygotes can be used for microinjection any time after pronuclei formation and expanding. Therefore, our streamlined procedures including IVF, vitrification/warming, optimized *in vitro* culture, and microinjection can be reproduced in any facility, enabling robust and efficient production of various knock-in mice.

## MATERIALS AND METHODS

### gRNA synthesis, construction of CRIS-PITCh (v2) vector, and preparation of Cas9 protein and ssODN

*In vitro* transcribed gRNAs were prepared according to a previous report (Aida et al., 2015). Briefly, template DNA fragments were generated using PCR amplification from CRISPR-Cas9 vectors with primers containing a T7 promoter sequence according to a previous protocol (Nakagawa et al., 2016). Subsequently, the gRNAs were synthesized using a MEGAshortscript T7 Kit (Life Technologies, Carlsbad, CA, USA), and then purified with a MEGAclear Kit (Life Technologies). For the CRIS-PITCh (v2) vector, a genomic region containing *Spp1* exons 3 and 4 was amplified from mouse genomic DNA and cloned into pGEM-3Z (Promega, Tokyo, Japan). Subsequently, base substitutions were introduced by site-directed mutagenesis. The two PITCh-gRNA target sites were then added to flank the microhomology sequences. The ssODNs were synthesized by Integrated DNA Technologies (Coralville, IA, USA). The sequences of oligonucleotides for gRNA templates, primers, and ssODNs are listed in Table S3. The recombinant Cas9 protein was obtained from New England Biolabs Japan (Cas9 Nuclease NLS, *Streptococcus pyogenes*; Tokyo, Japan) or Integrated DNA Technologies Japan (Alt-R™ S.p. Cas9 Nuclease 3NLS; Tokyo, Japan).

### Animals

C57BL/6J mice were purchased from CLEA Japan (Tokyo, Japan). After breeding, C57BL/6J female mice were used as oocyte donors at 10–14 weeks of age. C57BL/6J male mice over 12 weeks of age were used as sperm donors for IVF. ICR mice at 8–20 weeks of age were used as recipients of injected zygotes. All animals were housed under a 12 h dark:12 h light cycle (light from 07:00 to 19:00) at 22±1°C with *ad libitum* food and water. All animal experiments were approved by the Animal Care and Experimentation Committee of the Center for Animal Resources and Development, Kumamoto University, and were carried out in accordance with the approved guidelines.

### IVF and vitrification/warming of fertilized oocytes

The IVF and vitrification/warming procedures were described previously (Nakagawa et al., 2015, 2016). Cauda epididymides were obtained from C57BL/6 male mice over 12 weeks of age, and used as a source of sperm for IVF. C57BL/6 female mice were ultra-superovulated by intraperitoneal administration of IASe (0.1 ml IAS and 3.75 IU eCG, CARD HyperOva^®^; Kyudo, Saga, Japan), followed 48 h later by intraperitoneal administration of hCG (7.5 IU, Gonatropin; Aska Pharmaceutical, Tokyo, Japan) (Takeo and Nakagata, 2015). The cumulus-oocytes complexes and inseminated sperm after pre-incubation for 1.5 h were incubated at 37°C in 5% CO_2_ and 95% humidified air. After 2.5 h incubation, the inseminated oocytes were rinsed three times with human tubal fluid (HTF) medium. The generated fertilized oocytes were cryopreserved by a simple vitrification method 6.5 h after insemination (Nakagata et al., 2013; Nakao et al., 1997). At later time points, the cryopreserved oocytes were warmed, cultured in potassium simplex optimized medium with amino acids (KSOM-AA) for 2–7 h at 37°C in 5% CO_2_ and 95% humidified air, and used for microinjection.

### Basic procedure of microinjection

RNPs, *in vitro* transcribed gRNA(s) and Cas9 protein were mixed with ssODN or CRIS-PITCh (v2) vector in 0.1 TE buffer (Aida et al., 2015) for microinjection. For the generation of *Spp1* exon 5-modified mice, a mixture of 30 ng/µl gRNA and 0.75 µM Cas9 protein (New England Biolabs Japan) was injected with 10 ng/µl ssODN into the pronucleus. For the generation of *Tyr* single-base-substituted mice, a mixture of 20 ng/µl gRNA and 0.5 µM Cas9 protein (Integrated DNA Technologies Japan) was injected with 10 ng/µl ssODN into the pronucleus. For the generation of *Spp1* exon 3/4-modified mice, a mixture of 40 ng/µl gRNA (exon3-gRNA: 16 ng/µl, exon4-gRNA: 12 ng/µl, PITCh-gRNA: 12 ng/µl) and 1 µM Cas9 protein (New England Biolabs Japan) was injected with 5 ng/µl CRIS-PITCh (v2) vector into the pronucleus. To determine the concentration of injected reagents, we preliminary tested two kinds of RNP concentration based on our previous report (Nakagawa et al., 2016), then we chose the optimal or sufficient condition for each locus for the generation of ssODN knock-in mice. For the generation of PITCh knock-in mice, we initially performed single blastocyst assay and zygote transfer using 1.5 µM Cas9 protein, but the double-positive pup evaluated by RFLP was not obtained. Then, we changed to the lower concentration as described above. The injected oocytes were cultured in KSOM-AA at 37°C in 5% CO_2_ and 95% humidified air for about 1 h. Surviving oocytes were transferred to the oviducts of pseudopregnant ICR female mice.

### Detailed experimental workflow of microinjection and zygote transfer

When the microinjection into zygote was performed after 6–7 h culture, we worked as a team of two people. In the morning, one person (the operator #1) checked vaginal plugs of female mice mated with vasectomy male mice in the mouse breeding room, and the other one (the operator #2) warmed zygotes in the laboratory room. After zygote incubation, the operator #1 performed microinjection continuously, and the operator #2 transferred the zygotes from the culture dish to the injection dishes before microinjection, and from the injection dishes to the transfer dish after microinjection. After 30 min from the first microinjection, the operator #2 carried the injected zygotes to the surgery room, and then began zygote transfer after surgery preparation. When microinjection was finished, the operator #1 collected the injected zygotes and brought them to the surgery room. Injected zygotes were transferred to the oviducts of pseudopregnant ICR female mice in turn.

In the case of short time culture of zygotes prior to microinjection, the required number of zygotes was warmed at a convenient time after female vaginal plugs were checked in the morning. Subsequent procedures were the same as described above.

### Analysis of pups

Pup tail lysates were prepared by an alkaline lysis method and PCR was performed using KOD FX (Toyobo, Osaka, Japan) with each primer set. For the analysis of *Spp1* exon 5-modified mice, the *Spp1* ex5 F and R primers listed in Table S3 were used according to a previous report (Nakagawa et al., 2016). Each PCR product was subjected to automatic electrophoresis using MultiNA (Shimadzu Corporation, Kyoto, Japan) or agarose gel electrophoresis. The PCR products were purified and analyzed by RFLP analysis using *Bsm*FI (New England Biolabs Japan) and then the PCR products identified as positive were analyzed by direct sequencing using an ABI 3130 Genetic Analyzer (Life Technologies) with a BigDye Terminator v1.1 or v3.1 Cycle Sequencing Kit (Life Technologies). For analysis of the *Tyr* gene, pup tail lysates were analyzed by PCR with the *Tyr* F and R primers listed in Table S3. The PCR products were purified and analyzed by RFLP analyses using *Hpy*CH4III, and then direct sequencing was performed as described above. In some pups, multiple bands were detected by PCR; in these cases, each RFLP-positive band was cut out from the agarose gel, the DNA extracted and individually sequenced. For the analysis of *Spp1* exons 3 and 4, PCR amplification was carried out using the *Spp1* ex3-4 F and R primers listed in Table S3. Each PCR product was subjected to automatic electrophoresis using MultiNA. The PCR products were purified and analyzed by RFLP analyses using *Nar*I or *Dra*I (New England Biolabs Japan). The PCR products identified as positive were analyzed by direct sequencing. The PCR products of all intended mutations that were detected by direct sequencing were subcloned into a pTA2 vector using Target Clone-Plus (Toyobo), followed by DNA sequencing. Some founders were mated with male or female wild-type C57BL/6 and then pups were subjected to DNA sequencing analysis of the target site and transgene analysis.

### Transgene analysis of pups generated with the CRIS-PITCh (v2) system

To examine unintended genomic integration of vector backbone, each knock-in founder was subjected to genomic PCR using KOD FX with the Tg F and R primers listed in Table S3. The PCR products were analyzed by automatic electrophoresis using MultiNA.

### Off-target Analysis of pups generated with the CRIS-PITCh (v2) system

Candidate off-target sites for the gRNAs used for PITCh knock-in were selected using COSMID (https://crispr.bme.gatech.edu/), according to a previous protocol (Sakuma et al., 2017). Genomic regions flanking the top five potential off-target sites were amplified by PCR from knock-in founders using KOD FX with the primers listed in Table S3. The PCR products were analyzed by automatic electrophoresis using MultiNA and direct sequencing.
